# IL-17/Notch1/STAT3 Pathway Contributes to 5-Fluorouracil-Induced Intestinal Mucositis in Rats: Amelioration by Thymol Treatment

**DOI:** 10.3390/ph15111412

**Published:** 2022-11-15

**Authors:** Amira M. Badr, Layla A. Alkharashi, Iman O. Sherif, Alaa A. Alanteet, Hind N. Alotaibi, Yasmen F. Mahran

**Affiliations:** 1 Department of Pharmacology and Toxicology, College of Pharmacy, King Saud University, Riyadh 11211, Saudi Arabia; 2 Department of Pharmacology & Toxicology, Faculty of Pharmacy, Ain Shams University, Cairo 11566, Egypt; 3 Emergency Hospital, Faculty of Medicine, Mansoura University, Mansoura 35516, Egypt

**Keywords:** 5-fluorouracil, intestinal mucositis, thymol, Notch/STAT3 pathway

## Abstract

5-Fluorouracil (5-FU) is an anticancer drug with intestinal mucositis (IM) as a deleterious side effect. Thymol is a monoterpene phenol which has been reported to possess an antioxidant and anti-inflammatory activity versus 5-FU-induced IM. The Notch pathway affects multiple cellular activities, such as cellular proliferation, in addition to inflammatory responses modulation. Accordingly, this work was carried out in order to elucidate the role of the Notch pathway in 5-FU-induced IM and to further elucidate the immunomodulatory protective mechanisms of thymol. Experimental rats were divided randomly into four groups: Control, 5-FU, 5-FU+thymol (60 mg/kg/day), and 5-FU+thymol (120 mg/kg/day). 5-FU was injected intraperitoneally at a dose of 150 mg/kg on days 6 and 7, while thymol was orally administered daily for 11 days. By the end of the study, intestinal tissues were collected for the determination of IL-17, CD4, CD8, Notch1, Hes-1, pSTAT3, and STAT-3 protein expressions. The effect of thymol on 5-FU cytotoxicity was also examined using WST1 assay. 5-FU induced a marked increase in IL-17 levels, along with a marked downregulation of CD4 and the upregulation of CD8, Notch1, Hes-1 protein expressions, and activation of STAT3 in the intestinal tissue when compared with the control group. Thymol ameliorated the changes that occurred in these parameters. Additionally, cytotoxicity testing revealed that thymol augmented the antiproliferative action of 5-FU against breast and colorectal human cancer cell lines. This study was the first to show that the IL-17/Notch1/STAT3 pathway is involved in the molecular mechanism of 5-FU-induced IM, as well as the immunomodulatory activity of thymol.

## 1. Introduction

The 5-fluorouracil (5-FU) is an anticancer drug classified as an antimetabolite pyrimidine antagonist, and has been widely used against different organ malignancies. It is also considered the first line of treatment for colorectal cancer patients [1]. Cancer patients suffer from severe adverse effects associated with chemotherapy administration. One of these effects is chemotherapy-induced intestinal mucositis (IM), which is linked with the clinical use of 5-FU that, consequently, can lead to its discontinuation and increased hospitalization rates [2]. 

IM is characterized by gut injury in which the mucous membranes of the gastrointestinal tract are damaged with the loss of intestinal structure and function impairment, leading to pain, diarrhea, nausea, and vomiting [3]. Moreover, it has been reported that IM is associated with a storm of oxidative stress mediators and inflammatory cytokines which enhance intestinal injury and intestinal barrier rupture [1].

Additionally, the gastrointestinal tract’s mucosal homeostasis necessitates the participation of both innate and adaptive cells, such as macrophages and B and T lymphocytes, respectively [4]. The adaptive immune system relies heavily on CD4^+^ T cells. The majority of CD4^+^ T lymphocytes in the gut are found in the lamina propria. Naive CD4^+^ T cells can differentiate into one of four subtypes after being stimulated; T helper 1 (Th1) (producing interleukin IL-2 and interferon IFN-γ), Th2 (producing IL-4, IL-10), Th17 (producing IL-17A), or regulatory T cells (Treg) (producing IL-10 and TGF-β) [5]. Aberrant Th cell responses are implicated in the pathogenesis of inflammatory diseases [6].

Moreover, macrophages, immune cells derived from monocytes, are a major source of cytokines that are essential for the effective activation of T and B cells. Cytokines are now widely recognized as a critical player in immune response communication between T cells, macrophages, and other immune cells [7]. Two main types of macrophages were found. The M1 macrophage aggravates inflammation and barrier damage via secretion of pro-inflammatory cytokines, including IL-6 and TNF-α, which are capable of triggering T cell-mediated responses. On the other hand, the M2 macrophage has the ability to suppress inflammation via releasing anti-inflammatory cytokines involving IL-10 [8]. Shifting macrophages from M1 to M2 minimizes inflammation and barrier reconstruction [9].

On the other hand, interleukin-17 (IL-17) is a main pro-inflammatory cytokine produced mostly by CD4 T-helper 17 (Th17) cells in addition to other cells, including macrophages, dendritic cells, and natural killer cells [10]. It has been documented that IL-17 plays a crucial role in initiating and maintaining inflammation, as it has a role in chemotaxis induction as well as inflammatory cells accumulation at inflammation sites [11]. 

It has been reported that epithelial cell homeostasis is regulated by the Notch signaling system, which is important for epithelial integrity because it plays a role in regulating the balance of secretory and absorptive cell lineages, in addition to promoting epithelial cell proliferation [12]. The Notch signaling pathway regulates the differentiation and activation of various immune cell types in which activation of Notch triggers a pro-inflammatory action, and its blocking may be a promising tool for intestinal inflammation treatment [13]. 

Moreover, crosstalk of IL-17 with Notch1 has been reported to upregulate Th17-induced Notch1 target genes, which are included in the processes of inflammation and cell proliferation [14]. Additionally, the differentiation and regulation of Th17 cells and macrophages were documented to be linked to the STAT3 pathway. Therefore, STAT3 was considered a vital mediator in immune cell development and inflammation regulation [15]. 

There have been many attempts to ameliorate the severity of chemotherapy induced IM through using antiemetic palonosetron, metoclopramide, and diosmectite. However, the advances have not significantly reduced the heavy burden of chemotherapeutic drug toxicity. Recently, a growing number of studies have focused on combinations of drugs, natural compounds, and fecal microbiota transplantation to overcome the side effects of the chemotherapeutic agents [16,17]. Thymol, a natural phenol derivative with a chemical name of 5-methyl-2-isopropylphenol, is a carvacrol isomer and the main component in thyme oil, which is extracted from *Thymus vulgaris* [18]. Thymol has various biological actions, including antioxidant and anti-inflammatory properties, in addition to antitumor effects [19]. 

Our team reported previously the cytoprotective activity of thymol against 5-FU induced-IM through suppressing the inflammatory mediators: nuclear factor-kappa B (NF-kB), tumor necrosis factor-α (TNF-α), IL-6, cyclooxygenase-2 (COX-2), and prostaglandin (PGE2) associated with increasing the anti-inflammatory cytokine IL-10 level, in addition to downregulating the TGF-β/p38/p-JNK signaling pathway in the small intestine [20]. Therefore, we extended our work in order to elucidate, in depth, the pathophysiological mechanism of 5-FU induced-IM, as well as the molecular mechanism of the immunomodulatory action of thymol against 5-FU induced-IM through the IL-17/Notch1/STAT3 signaling pathway.

## 2. Results

### 2.1. Impact of Thymol Administration on the Histopathological Features of Intestinal Samples of 5-FU-Intoxicated Rats

**Figure 1:**
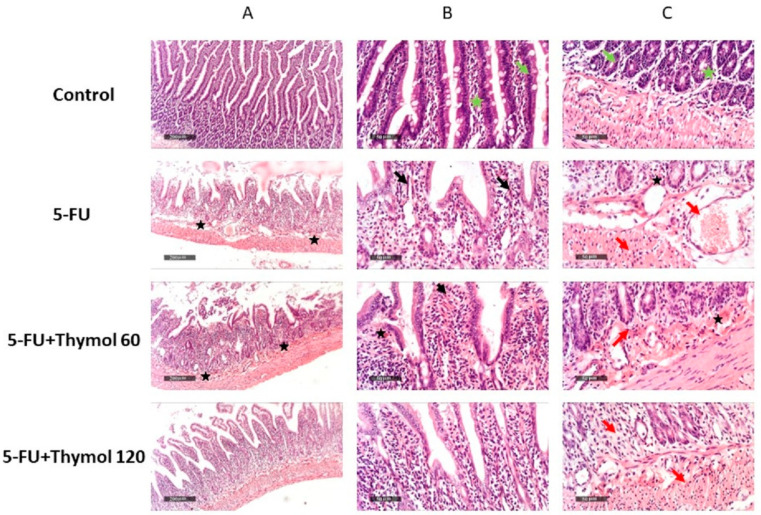
Impact of thymol administration on the histopathological features of intestinal samples of 5-FU-intoxicated rats. Control (**A**–**C**): normal control rats; FU (**A**–**C**): untreated 5-FU rats; FU + Thymol 60 (**A**–**C**): 5-FU rats treated with thymol (60 mg/kg/day, p.o.); FU + Thymol 120 (**A**–**C**): 5-FU rats treated with thymol (120 mg/kg/day, p.o.). Control group showed normal histological structure of the intestinal mucosa. 5-FU treated group showed severe atrophy and fusion of intestinal villi (arrow), with significant mucosal mixed inflammatory cell infiltrates (red arrow) and many dilated and congested submucosal blood vessels (star). FU+ Thymol 60 group did not show any amelioration and demonstrated almost the same records as 5-FU samples, without significant improvement of morphological features. FU+ thymol 120 group showed moderate protective efficacy, with restoration of intestinal villi of normal length and morphology with some records of mucosal inflammatory cell infiltrates (red arrow), as well as minimal congested blood vessels.

In order to understand the impact of thymol on 5-FU-induced intestinal mucositis, we examined the morphological features of intestinal mucosa in the different treatment groups. Figure 1 (Control A–C) shows the control intestinal tissue samples that demonstrated normal morphological features of the intestinal mucosa, including intact intestinal villi and crypts with apparent intact enterocytes showing intact subcellular details (green arrow) (Control B, C). Minimal inflammatory cell infiltrates were shown in submucosa with intact vasculatures (green star) (Control B, C). On the other hand, 5-FU specimens showed severe atrophy and fusion of intestinal villi (black arrow) (5-FU B), with significant mucosal mixed inflammatory cell infiltrates (red arrow) (5-FU C) and many dilated and congested submucosal blood vessels (star) (5-FU C). Administration of thymol 60 mg did not show any amelioration, and demonstrated almost the same records as 5-FU samples without significant improvement of morphological features (FU + Thymol 60 A–C). Samples treated with 120 mg of thymol demonstrated moderate protective efficacy with the restoration of intestinal villi of normal length, morphology with some records of mucosal inflammatory cells infiltrates (red arrow), and minimal congested blood vessels, Figure 1 (FU + Thymol 120 C).

### 2.2. Thymol Administration Suppresses the Intestinal Expression of IL-17 in the 5-FU-Intoxicated Rats

**Figure 2:**
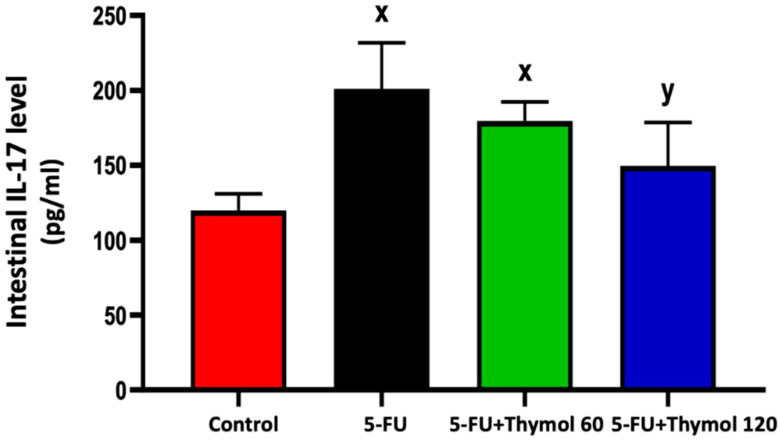
Impact of thymol oral administration (60 and 120 mg/kg/day) on intestinal IL-17 levels in 5-FU-intoxicated rats. Data are presented as mean ± SD. ^x^ *p* < 0.05 versus control group, ^y^ *p* < 0.05 versus 5-FU group.

Next, we sought to understand the role of thymol on 5-FU intoxicated rats at the molecular level. Therefore, we immunostained intestinal paraffin-embedded sections from each treatment group with IL-17. Our finding demonstrated a significant rise in the intestinal IL-17 level by 67.6% in the 5-FU-intoxicated rats compared with normal control rats (Figure 2). However, a marked decline in the intestinal IL-17 level was observed after treatment with 120 mg/kg thymol by 25.55% when compared with the 5-FU injected rats (*p* < 0.05). 

### 2.3. Thymol Administration Induced CD4 Intestinal Expression in the 5-FU-Intoxicated Rats

**Figure 3:**
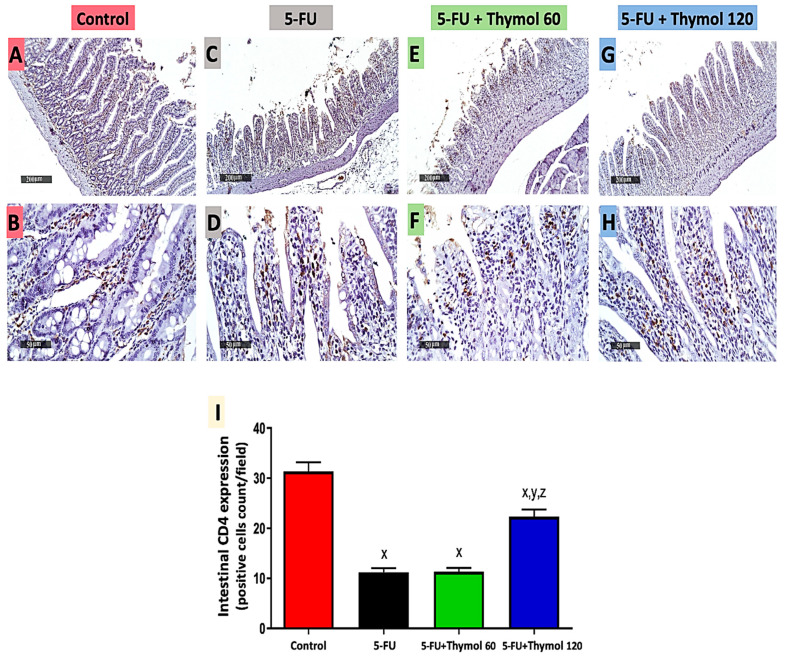
Microscopic pictures of intestinal CD4 expression in all experimental groups by immunohistochemical staining (**A**–**H**) and its statistical analysis as positive cell count/field (**I**). Intestinal sections showed an extensive CD4 expression in the control group (**A**,**B**), minimal expressions in 5-FU (**C**,**D**) and 5-FU + Thymol 60 (**E**,**F**), and increased expression in 5-FU + Thymol 120 (**G**,**H**). (**A**,**C**,**E**,**G**): ×200, bar 200 μm and (**B**,**D**,**F**,**H**): ×400, bar 50 μm. Data are presented as mean ± SD. ^x^ *p* < 0.05 versus control group, ^y^ *p* < 0.05 versus 5-FU group, ^z^ *p* < 0.05 versus 5-FU + thymol 60 group.

Following this, we investigated the impact of thymol administration on intestinal CD4 expression in 5-FU intoxicated rats. As illustrated in Figure 3, immunostained intestinal sections against CD4 showed positive brown staining in the control group (Figure 3A,B) and low brown staining in both 5-FU injected rats (Figure 3C,D) and the thymol 60 group (Figure 3E,F). A rise in the positive brown staining was observed in the thymol 120 group (Figure 3G,H). Furthermore, the statistical scores of the intestinal CD4 expression showed significant downregulation in intestinal CD4 expression in the 5-FU group, by 64.2% in comparison to the control group, while a marked upregulation was detected in the thymol 120 group by 1-fold when compared with the 5-FU group. Furthermore, rats administered 120 mg/kg of thymol showed a significant upregulation in CD4 intestinal expression by 1-fold compared with the 60 mg/kg thymol group (*p* < 0.05).

### 2.4. Impact of Thymol Administration on CD8 Intestinal Expression and CD4/CD8 Ratio in the 5-FU-Intoxicated Rats

**Figure 4:**
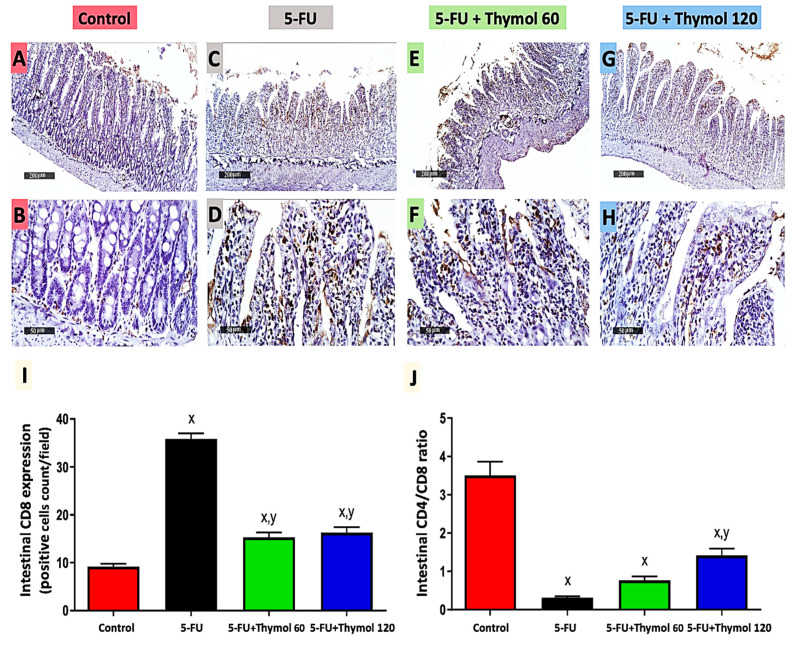
Microscopic pictures of intestinal CD8 expression in all experimental groups by immunohistochemical staining (**A**–**H**) and its statistical analysis as positive cell count/field (**I**). Intestinal sections showed a minimal CD8 expression in the control group (**A**,**B**), extensive expression in the 5-FU (**C**,**D**) group, and a moderate expression in the 5-FU + Thymol 60 (**E**,**F**) and 5-FU + Thymol 120 (**G**,**H**) groups. Intestinal CD4/CD8 ratio (**J**). (**A**,**C**,**E**,**G**): ×200, bar 200 μm and (**B**,**D**,**F**,**H**): ×400, bar 50 μm. Data are presented as mean ± SD. ^x^ *p* < 0.05 versus control group, ^y^ *p* < 0.05 versus 5-FU group.

Further evidence supporting the beneficial effects of thymol administration on 5-FU intoxicated rats is demonstrated in Figure 4. The immunostained intestinal sections against CD8 displayed mild brown staining in the control group (Figure 4A,B), while showing intense brown staining in 5-FU (Figure 4C,D), where a significant upregulation of CD8 expression was found in the 5-FU group (2.9 fold) in comparison to the control group. However, low positive brown staining was observed in both the thymol 60 (Figure 4E,F) and thymol 120 (Figure 4G,H) groups. This showed a significant downregulation of CD8 expression by 57.3% and 54.5%, respectively, when compared with 5-FU group (*p* < 0.05). Furthermore, 5-FU induced a notable decrease of 91.4% in the intestinal CD4/CD8 ratio when compared with the control group. However, the thymol 120 treatment revealed a marked rise in the intestinal CD4/CD8 ratio by 3.7-fold compared to the 5-FU-intoxicated group, *p* < 0.05 (J).

### 2.5. Impact of Thymol Administration on Notch1 Intestinal Expression in the 5-FU-Intoxicated Rats

**Figure 5:**
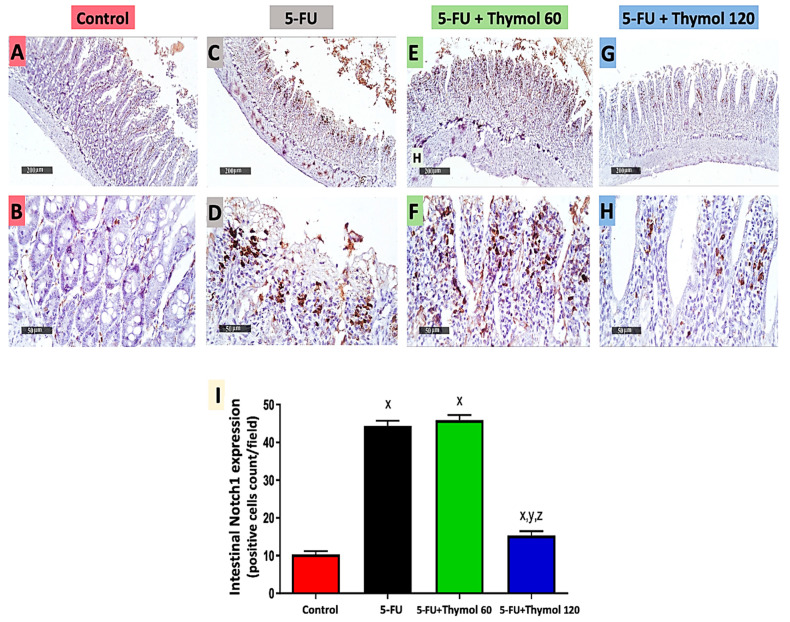
Microscopic pictures of intestinal Notch1 expression in all experimental groups by immunohistochemical staining (**A**–**H**) and its statistical analysis as positive cell count/field (**I**). Intestinal sections showed a minimal Notch expression in the control group (**A**,**B**), an extensive expression in the 5-FU (**C**,**D**) and 5-FU + Thymol 60 (**E**,**F**) groups, and a minimal expression in the 5-FU + Thymol 120 (**G**,**H**) groups. (**A**,**C**,**E**,**G**): ×100, bar 200 μm and (**B**,**D**,**F**,**H**): ×50, bar 50 μm. Data are presented as mean ± SD. ^x^ *p* < 0.05 versus control group, ^y^ *p* < 0.05 versus 5-FU group, ^z^ *p* < 0.05 versus 5-FU + thymol 60 group.

Next, we examined the impact of thymol on the intestinal expression level of Notch1. As illustrated in Figure 5, immunostained intestinal sections against Notch1 showed positive brown staining in the control group (Figure 5A,B) and intense brown staining in both 5-FU injected rats (Figure 5C,D) and the thymol 60 group (Figure 5E,F). However, low staining was observed in the thymol 120 group (Figure 5G,H). Moreover, the statistical scores of intestinal Notch1 expression showed a marked upregulation in intestinal Notch1 expression in the 5-FU group by 3.3-fold in comparison to the control group, while a significant downregulation was observed in the thymol 120 group by 65.5% when compared with the 5-FU group. Furthermore, the thymol 120 group displayed a marked downregulation in intestinal Notch1 expression by 66.6% in comparison to the thymol 60 group (*p* < 0.05).

### 2.6. Impact of Thymol Administration on Hes-1 Intestinal Expression in the 5-FU-Intoxicated Rats

**Figure 6:**
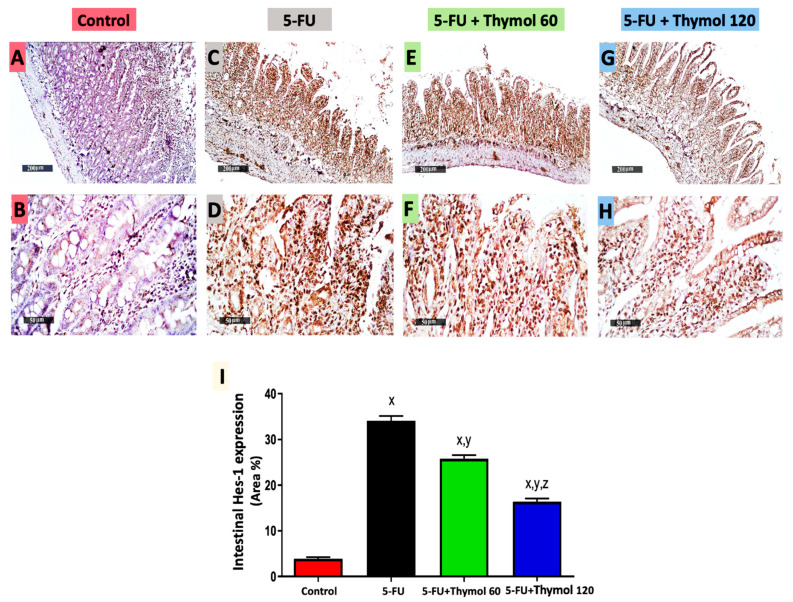
Microscopic pictures of intestinal Hes-1 expression in all experimental groups by immunohistochemical staining (**A**–**H**) and its statistical analysis as area % of immune-positive staining (**I**). Intestinal sections showed a minimal Hes1 expression in the control group (**A**,**B**), an extensive expression in the 5-FU (**C**,**D**) and 5-FU + Thymol 60 (**E**,**F**) groups, and a moderate expression in the 5-FU + Thymol 120 (**G**,**H**) groups. (**A**,**C**,**E**,**G**): ×200, bar 200 μm and (**B**,**D**,**F**,**H**): ×400, bar 50 μm. Data are presented as mean ± SD. ^x^ *p* < 0.05 versus control group, ^y^ *p* < 0.05 versus 5-FU group, ^z^ *p* < 0.05 versus 5-FU + thymol 60 group.

Additionally, Figure 6 displays immunostained intestinal sections against Hes-1, which showed positive brown staining in the control group (Figure 6A,B) and intense brown staining in both the 5-FU (Figure 6C,D) and thymol 60 groups (Figure 6E,F). However, moderate staining was observed in the thymol 120 group (Figure 6G,H). Moreover, the statistical scores of intestinal Hes-1 expression displayed a significant upregulation in intestinal Hes-1 expression in the 5-FU group, by 7.7-fold in comparison to the control group. However, a significant downregulation was detected in the thymol 60 (by 24.3%) and thymol 120 (by 51.9%) groups when compared with the 5-FU group. Furthermore, the group treated with 120 mg/kg of thymol exhibited a marked downregulation of 36.4% in intestinal Hes-1 expression when compared with the group treated with 60 mg/kg of thymol (*p* < 0.05).

### 2.7. Impact of Thymol Administration on the p-STAT3/t-STAT3 Intestinal Ratio in the 5-FU-Intoxicated Rats

**Figure 7:**
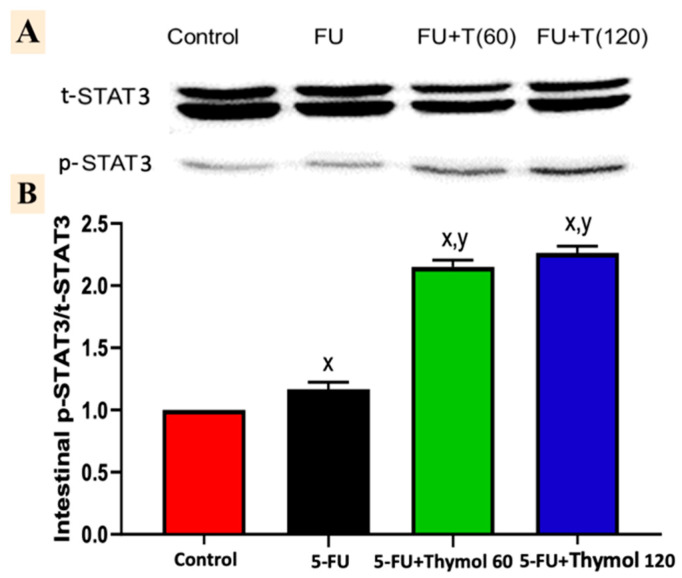
Impact of thymol oral administration (60 and 120 mg/kg/day) on the intestinal p-STAT3 and t-STAT3 proteins levels in 5-FU-intoxicated rats. The Western blot bands of p-STAT3 and t-STAT3 of protein levels (**A**), and the statistical analysis of the p-STAT3/t-STAT3 ratio (**B**). Data are presented as mean ± SD. ^x^ *p* < 0.05 versus control group, ^y^ *p* < 0.05 versus 5-FU group.

The injection of 5-FU induced a marked activation of intestinal p-STAT3, as shown in the Western blotting analysis. Figure 7A shows that the intestinal p-STAT3/t-STAT3 ratio was significantly elevated by 16.7% after 5-FU injection, in comparison to the control group. However, administration of thymol in doses of 60 mg and 120 mg showed a marked increase in the intestinal p-STAT3/t-STAT3 ratio of 84.2% and 93.9%, respectively, when compared with 5-FU intoxicated rats (*p* < 0.05).

### 2.8. Thymol Augments the Cytotoxic Effect of 5-FU in Human Cancer Cells

**Figure 8:**
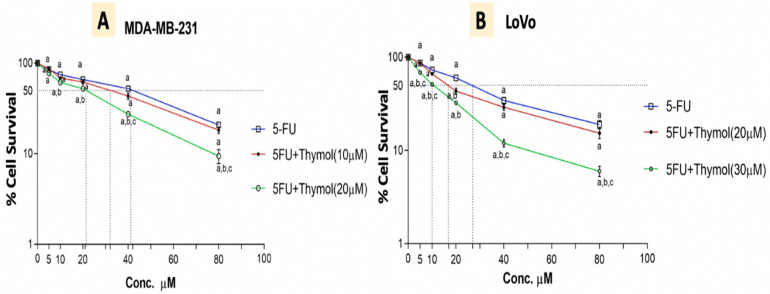
WST1 cell cytotoxicity assay of cell survival for MDA-MB-231 (**A**) and LoVo (**B**) cells treated for 48 h with either 5-FU alone or in a sequential combination with thymol at different concentrations. Results are expressed as means ± SD. ^a^ *p* < 0.05 versus untreated control cells, ^b^ *p* < 0.05 versus the same concentration of 5-FU, ^c^ *p* < 0.05 versus the same concentration of 5-FU and lower dose of thymol.

Data from our study guided us to test the feasibility of thymol inducing 5-FU cytotoxicity in human cancer cells. Therefore, cell cytotoxicity was assessed using the WST1 assay, and cell viability was expressed in terms of the survival fraction relative to the non-treated control cells, as illustrated in Figure 8. Treatment with 5-FU for 48 hours induced a significant cytotoxicity in both MDA-MB-231 “breast” and LoVo “colon” human cancer cell lines, in a dose-dependent manner. The IC50 of 5-FU was found to be 41 and 28 μM for MDA-MB-231 and LoVo cells, respectively. However, treatment with thymol significantly increased the 5-FU cytotoxicity in both cells in a dose-dependent manner, whereas using thymol at a concentration of 10 and 20 μM reduced the 5-FU IC50 significantly to 30 and 21 μM for the MDA-MB-231, respectively. Additionally, treatment of thymol with 20 and 30 μM reduced the IC50 of 5-FU to approximately 17 and 10 μM for LoVo cells, respectively.

## 3. Discussion

5-FU is a type of chemotherapy indicated for the treatment of various types of malignancies, including gastrointestinal malignancy. Upon its administration, patients may suffer from oral mucositis and IM as a drawback, worsening patients’ life quality. This could lead to the discontinuation of the chemotherapy [2,21].

It has been reported that the immune cell responses were implicated in the pathogenesis of chemotherapy-induced IM. Activated effector T-helper cells (Th17) released a storm of pro-inflammatory cytokines and triggered a flare-up of inflammation, causing damage to the intestinal mucosa [22]. One of these inflammatory mediators is IL-17, in which the mRNA expression was reported to be upregulated in the mesenteric lymph node in rats following chemotherapy-induced IM [22], as well as in the inflamed mucosa of patients suffering from inflammatory bowel disease [23]. However, a marked increase in IL-17 intestinal protein levels following a 5-FU injection was documented in our study.

Moreover, our results showed that 5-FU induced a marked downregulation in CD4 and upregulation of CD8, associated with a decreased CD4/CD8 ratio, when compared with the control group. The release of cytokines, which manage the amplitude and duration of both immunological and inflammatory responses, is a primary role of T cells. CD4^+^ and CD8^+^ T cells produce IL-2, IFN-γ, and TNF-α, which enhance inflammation, while CD4^+^ Th2 cells produce IL-10 and IL-4, which inhibit these responses [7]. Additionally, IL-17 is known to activate NF-kB and cooperate with TNF-α inducing inflammatory cytokines [7] and our previous work reported a marked rise in the intestinal levels of IL-6 and TNF-α along with a significant decline in the intestinal levels of IL-10 following 5-FU injection [20]. 

Notch proteins act as receptors for transmembrane ligands. Notch receptors are proteolytically cleaved by γ-secretase after ligand activation, releasing the Notch intracellular domain (NICD), which is translocated into the nucleus, forming a transcriptional activator complex that activates the Notch target genes as Hes1 in the intestinal mucosa [12]. Moreover, it has been reported previously that Notch signaling within the intestinal epithelium is upregulated in the inflamed mucosa in patients with ulcerative colitis, as well as in dextran sodium sulfate-treated mice [24]. In parallel, our results showed upregulation in Notch and Hes1 intestinal expression following 5-FU administration. 

Previous research documented that Notch signaling activates kinases for STAT3 phosphorylation and activation. Alternatively, the target gene of Notch signaling Hes-1 was determined to interact with STAT3 and to enhance the STAT3 phosphorylation and activation [25,26]. STAT3 is a member of the STAT (signal transducer and activator of transcription) family of proteins. STAT3, like the other STAT proteins, is sequestered in an inactive form in the cytoplasm. When activated, it translocates into the nucleus, where it acts as a transcription activator for a wide range of genes. Phosphorylation of a crucial tyrosine residue (Tyr 705) causes STAT3 activation, which is predominantly regulated by Janus-activated kinases (JAK) as a key modulator [27]. 

Recently, it has been documented that the JAK/STAT3 pathway is implicated in the pathogenesis of intestinal mucosal inflammation involving 5-FU-induced IM [28]. In our study, intestinal p-STAT3 expression was markedly activated following a 5-FU injection. Similarly, mice injected with 5-FU exhibited a marked upregulation in p-STAT3 protein expression in colon tissue [28]. Moreover, in 2015, Yuan and his colleagues reported that IL-17 stimulates STAT3 activation, in which Tyr 705 phosphorylation is important for IL-17’s mediation of the activation of STAT3 [15]. 

In order to combat chemotherapy-induced IM, effective therapies are required [2]. Thymol has been studied in recent years for its anti-inflammatory, antioxidant, and immunomodulatory properties. Its toxicological profile showed no effect on normal tissues in the selected dose [29]. The inhibitory effects of thymol on the immunological responses, as well as various cells and components of the immune system, were reported in several investigations [30]. One study documented the suppressive effect of thymol on IL-17 levels in the splenocyte culture and serum of ovalbumin-immunized mice [6]. This finding was in line with our results, in which a significant decline in intestinal IL-17 was detected after thymol treatment. Additionally, the immunomodulatory effect of thymol via enhancing CD4 expression in mice treated with cyclosporine-A was documented, and coincided with our findings [31]. We suggested that downregulation of the CD8 intestinal expression might be implicated in the anti-inflammatory action of thymol, which was previously reported [20,32] to act against the CD8-mediated cytotoxic activity and exaggerated inflammation [33]. 

Notch signaling activated the production of the pro-inflammatory cytokine IL-6 via the NF-kB pathway in colonic epithelium, and the blockade of the Notch pathway resulted in a reduction of intestinal inflammation in a model of trinitrobenzene sulfonic acid-induced colitis in mice [34]. Therefore, inhibiting the Notch pathway could be a novel approach for minimizing inflammation by interfering with M1 macrophage cytokine production and Th1 cell infiltration [33]. Thymol exhibited a marked downregulation of Notch1 and Hes-1 intestinal expression in our study. Carvacrol, a thymol isomer, showed anti-inflammatory [32] and antitumor activity [35]. Its antitumor activity was reported to be related to its inhibition of Notch signaling through Notch-1 downregulation in prostate cancer cells (PC-3) [35]. 

Interestingly, our results revealed that thymol administration induced a marked upregulation of p-STAT3/ STAT3 levels. Although it has been reported that thymol contributed to minimizing the inflammatory responses via decreasing the levels of p-STAT3 in lipopolysaccharide-treated macrophages [36], luteolin, another natural compound, has recently showed a cardioprotective effect by inhibiting inflammatory reactions via activation of STAT3 signaling in a myocardial ischemia/reperfusion model [37]. 

On the other hand, the antiproliferative action of thymol against MDA-MB 231 [38] and LoVo [39] has been previously documented. It has been reported that the adjuvant use of natural products with chemotherapy will result in potentiating the action of the chemotherapy [40]. In the current study, the cytotoxicity assay performed showed that thymol augmented the antiproliferative action of 5-FU against breast and colorectal human cancer cell lines. 

## 4. Materials and Methods

### 4.1. In Vivo Study

#### 4.1.1. Experimental Animals and Design

Thirty-two male Wistar rats (7 weeks old, 150–200 g) were provided from the animal house of the Faculty of Pharmacy, King Saud University, Riyadh, Saudi Arabia, and were kept for acclimatization for 7 days before starting the experiment at the animal house. They were kept in standard cages under controlled conditions (25 °C, 12-h light/dark cycles) and permitted free access to a standard diet and water ad libitum. The experimental protocol was reviewed and approved by the ethical committee at College of Pharmacy, King Saud University, Riyadh, Saudi Arabia, with the approval number SE-19-154.

Rats were randomly divided into four groups, in which each group consisted of 8 rats, and were treated for 11 days as described in Table 1. The doses used for 5-FU and thymol were selected according to our previous study [20].

#### 4.1.2. Samples Collection

On day 11, rats were sacrificed by decapitation under anesthesia. Intestinal tissues were dissected and then washed with ice-cold PBS. For histopathology and immunohistochemistry work, part of the small intestine (mainly jejunum) was preserved in 10% formalin solution; however, another part of the intestinal tissue was snap-frozen in liquid nitrogen, then kept at −80 °C for further analysis. Furthermore, the intestinal tissue homogenate was prepared by homogenization with PBS and centrifuged at 4 °C for 15 min at 10,000 rpm. Supernatants were collected and kept at −80 °C until utilization.

#### 4.1.3. Histopathological Examination

Intestinal tissue was fixed in formalin solution, dehydrated, cleared in xylene, and then placed in paraffin. Sections with a thickness of 5 μm were cut, prepared for hematoxylin and eosin (H&E) staining, and examined under a light microscope by a blinded pathologist. 

#### 4.1.4. ELISA Assessment of IL-17

Levels of IL-17 were assessed in the intestinal tissue homogenate using a commercially available rat ELISA kit (Elabscience Biotechnology Co., Wuhan, China), in accordance with the manufacturer’s instructions. 

#### 4.1.5. Immunohistochemical Detection of CD4, CD8, Notch, and Hes-1, Expressions

Immunohistochemistry (IHC) examination was performed according to a standard protocol, in which the intestinal paraffin blocks were briefly deparaffinized, rehydrated, and incubated with primary antibodies against CD4 (1:100), CD8 (1:100), Notch1 (1:100), and Hes-1 (1:100), which were purchased from Abcam, Inc., Cambridge, UK, and followed by appropriate secondary antibodies. Incubation with diaminobenzidine (DAB) and counterstaining with Mayer’s hematoxylin were carried out for the purpose of visualizing slides under a light microscope. Histopathological scores for CD4, CD8, and Notch1 were calculated as positive cell counts per field, while the scores for Hes-1 were calculated as area % of the intestinal immunopositive expression of Hes1, in which 6 random fields/section were used.

#### 4.1.6. Immunoblot Analysis of Phosphorylated-STAT3/STAT3

An intestinal tissue homogenate was mixed with protease/phosphatase inhibitor cocktails, and the quantitative protein analysis was conducted by Direct Detect quantification. Intestinal protein was extracted from 60 µm of the intestine (SDS-PAGE) and electroblotted onto PVDF membranes. The membranes were further incubated at room temperature for 1 h with a 5% mixture of nonfat dry milk and BSA, followed by incubation at 4 °C with Tris-buffer saline, Tween, and primary antibodies of p-STAT3 (1:1000, cat. #9131) and STAT3 (1:100, cat. #30835) from cell signaling. Membranes were then washed and incubated with a secondary antibody anti-rabbit HRP-conjugate (1:5000). The immunoblot densitometric analysis was carried out in order to quantify the quantity of the studied protein (p-STAT3) against the control sample by total protein (t-STAT3) normalization. An enhanced chemiluminescence detection kit (GE Healthcare) was used for blotting proteins, and Image Quant LAS 4000 mini was used for visualizing the immunoreactive bands. Image J software (Version 1.8.0, Bethesda, MD, USA) was used for densitometric analysis.

### 4.2. In Vitro Study

#### 4.2.1. Cell Lines

Human breast adenocarcinoma MDA-MB-231 and colon cancer LoVo cell lines (ATCC, Manassas, VA, USA) were cultured with DMEM containing 5% FBS and 1% of antibiotic/Antimycotic (GIBCO^®^, InvitrogenTM, Carlsbad, CA, USA), and incubated with 95% of relative humidity under 5% CO_2_ at 37 °C.

#### 4.2.2. Cytotoxicity WST-1 Assay

The WST-1 assay was used to measure the in vitro cytotoxic effects of 5-FU, either alone or in combination with thymol, on MDA-MB-231 “Triple-negative breast cancer” and LoVo “human colon cancer.” In brief, 5000 cells/well were seeded into 96-well microtiter plates with 100 µL of DMEM supplemented medium, and incubated overnight. Following this, the appropriate drug concentrations were added and incubated for 48 h. 5-FU was used at different concentrations between 5 and 80 μm. Thymol was used at a concentration of 10 and 20M for MDA-MB-231 and at a concentration of 20 and 30 µM for LoVo. After treatment, cells were fixed with 10 µL of WST-1 reagent (Abcam, Cambridge, UK) and incubated at 37 °C for 4 h. Blank control wells contained 100 µL of culture medium and 10 µL of WST-1. Formazan dye optical density was measured spectrophotometrically at 440 nm for each well. The % cytotoxicity was calculated as % *Cytotoxicity* = 100 × [(treated − blank)/(control − blank)] A concentration–response curve was sketched, and the inhibitory concentration 50 (IC 50) was determined for each curve (Graph Pad, Prism software). 

### 4.3. Statistical Analysis

The statistics of this study were assessed by using GraphPad Prism software, version 8. One-way ANOVA, followed by Tukey’s post-hoc test, was used for comparisons among groups. Data were represented as mean ± SD. When *p* < 0.05, results were considered to be statistically significant. 

## 5. Conclusions

Our study suggested that thymol has an immunomodulatory effect in the model of 5-FU-induced IM by exerting a significant reduction in IL-17 levels; downregulating CD8, Notch1, and Hes-1 protein expressions while upregulating CD4 protein expression; and activating pSTAT3 protein levels in intestinal tissue. This work investigated, for the first time, the involvement of the IL-17/Notch1/STAT3 signaling pathway in the immunomodulatory activity of thymol against 5-FU-induced IM. Additional studies are encouraged to validate our findings. 

## Figures and Tables

**Table 1:** Experimental groups used in the study and its description.

Groups	Description
Control	Rats received 0.5% dimethyl sulfoxide (DMSO, Sigma-Aldrich, St. Louis, MO, USA) daily by oral gavage.
5-FU	Rats received 0.5% DMSO daily by oral gavage plus 150 mg/kg 5-fluorouracil (5-FU, 50 mg/mL ampoules, Ebewe Pharma, Austria) injected intraperitoneally (i.p.) on the 6th and 7th days to induce intestinal toxicity.
5-FU + Thymol 60	Rats received 60 mg/kg/day thymol (Purity > 98%, Abcam, Cambridge, UK) in 0.5% DMSO orally, plus 150 mg/kg 5-FU injected i.p. on the 6th and 7th days.
5-FU + Thymol 120	Rats received 120 mg/kg/day thymol in 0.5% DMSO orally, plus 150 mg/kg 5-FU injected i.p. on the 6th and 7th days.

## Data Availability

Data is contained within the article.
